# Thermally Healable and Recyclable Graphene-Nanoplate/Epoxy Composites Via an In-Situ Diels-Alder Reaction on the Graphene-Nanoplate Surface

**DOI:** 10.3390/polym11061057

**Published:** 2019-06-18

**Authors:** Cho-Rong Oh, Dae-Il Lee, Jun-Hong Park, Dai-Soo Lee

**Affiliations:** 1 Division of Semiconductor and Chemical Engineering, Chonbuk National University, Baekjedaero 567, Jeonju 54896, Korea; ohcho38@naver.com (C.-R.O.); ldi4084@naver.com (D.-I.L.); 2 R & D Center, Lotte Advanced Materials, Sandan-ro 334-27, Yeosu 59616, Korea

**Keywords:** graphene-nanoplate, Diels-Alder reaction, epoxy composites, thermally healing

## Abstract

In this work, thermally healable graphene-nanoplate/epoxy (GNP/EP) nanocomposites were investigated. GNPs were used as reinforcement and crosslinking platforms for the diglycidyl ether of bisphenol A-based epoxy resin (DGEBA) through the Diels-Alder (DA) reaction with furfurylamine (FA). The GNPs and FA could then be used as a derivative of diene and dienophile in the DA reaction. It was expected that the combination of GNPs and FA in DGEBA would produce composites based on the interfacial properties of the components. We confirmed the DA reaction of GNPs and FA at the interface during curing of the GNP/EP nanocomposites. This procedure is simple and solvent-free. DA and retro DA reactions of the obtained composites were demonstrated, and the thermal healing properties were evaluated. The behavior of the GNP/EP nanocomposites in the DA reaction is similar to that of thermosetting polymers at low temperatures due to crosslinking by the DA reaction, and the nanocomposites can be recycled by a retro DA reaction at high temperatures.

## 1. Introduction

**Scheme 1:**
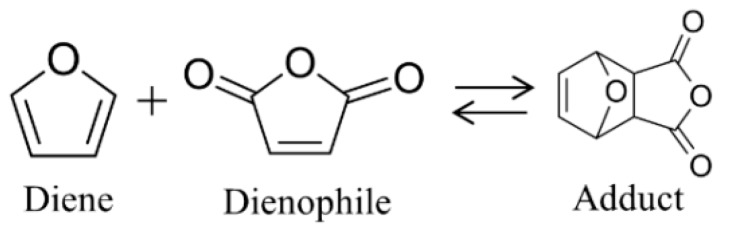
The Diels-Alder reaction between diene and dienophile.

Thermosetting polymers are widely used because of their good thermal stability, chemical resistance, and high mechanical strength. However, the reprocessing or recycling of thermosetting polymers is limited due to their molecular network architecture. Recently, numerous researchers reported self-healing and thermally remendable network polymers based on the dynamic covalent bonds formed via the Diels-Alder (DA) reaction, the metathesis of disulfide or imine, and the ester exchange reaction [1,2,3,4]. The DA reaction is a cycloaddition reaction between diene and dienophile, and the adduct is reversible at temperatures above 90 °C, as shown in Scheme 1**.**

Generally, a furfuryl group is used as the diene, whereas a maleimide group is used as the dienophile based on various potential modifications. The side reaction of maleimide can continue when the system is exposed to high temperatures in the presence of primary or secondary amine [5,6,7]. In most studies, two methods are used to introduce the DA reaction in an epoxy resin. First, to introduce the DA reaction in the epoxy backbone, the synthesis of epoxy or amine-terminated DA adduct is needed to protect the double bond of dienophile [8,9,10]. Second, a furan-terminated backbone polymer is prepared and then reacted with bismaleimide as a crosslinker in a solvent or using extrusion [11,12,13,14,15,16,17]. If the dienophile could be replaced with a material other than maleic anhydride or maleimide, then epoxy composites could be prepared by the DA reaction using a simple process.

Graphite is a commonly available material and is composed of multiple layers of graphene, *sp^2^* bonded carbon atoms arranged in a hexagonal, or honeycomb lattice. Graphene fabricated through various exfoliation methods [18,19], has exceptional electrical, thermal, and mechanical properties, and allows for a substantial enhancement of polymer properties at a much lower content than conventional fillers [20,21]. Graphitic fillers are used to impart self-healing properties based on π-π interactions or surface functionalization [22,23]. Furthermore, graphene can be used as a diene and dienophile in the DA reaction without modification. Sarkar et al recently proposed the use of single-layer graphene in DA and retro DA reactions as a dienophile or diene with another reactant at various temperatures [24]. Several studies reported the successful preparation of graphene nanoplates (GNPs), i.e., multilayered graphene, via the DA reaction of TCNE and graphite, and synthesized graphene oxide through a DA reaction between GNPs and maleic anhydride [25,26]. The DA reaction between a carbon-based material and mono-molecules, as well as polymers, has also been investigated [27,28]. Bai et al reported the synthesis of crosslinked poly(styrene-b-butadiene-b-styrene) (SBS) via the DA reaction between C60 and furan-modified SBS and evaluated its thermal healing properties [29]. Kötteritzsch and colleagues converted poly(lauryl methacrylate) using anthracene moieties in the side chain with C60 and phenyl-C61-butyric acid methyl ester, thereby producing a self-healing polymeric material [30]. According to these studies, GNPs can replace maleic anhydride or maleimide in a DA reaction. Moreover, the physical properties and electrical conductivity of the composites can be enhanced due to the characteristics of GNPs.

In the above-mentioned studies, a solvent was used for the dispersion of the GNPs and synthesis of the DA adduct. However, solvent-casting methods are not effective because the mechanical properties of the material are degraded during the drying process. This method also has issues with volatile organic compounds during film formation by drying. To address the low dispersibility of GNPs, we employed a masterbatch method in which the GNPs are dispersed in a base resin [31,32].

To simplify the surface modification process for GNPs, an in-situ DA reaction should occur during curing of the epoxy resin to produce a crosslinked GNP/epoxy (GNP/EP) nanocomposite via a DA reaction. The interfacial tension is used to determine the interfacial wetting. To ensure contact between the GNPs and the reactant for the DA reaction, the interfacial tension between the GNPs and the reactant should be lower than the sum of the interfacial tension between the matrix resin and the reactant and between the GNPs and the matrix. The interfacial tension can be calculated from the surface tension of each material. For this purpose, the commonly used Wu and Owen–Went interfacial tension equations are applied, as given in Equations (1) and (2), respectively. These formulas consider the dispersion and polar components of interfacial tension in accordance with the modified form of Fowke’s equations [33,34,35,36] as follows:

(1) $$\gamma_{S-l}=\gamma_{S}+\gamma_{l}-\frac{4\gamma_{S}^{d}\gamma_{l}^{d}}{\gamma_{S}^{d}+\gamma_{l}^{d}}-\frac{4\gamma_{S}^{p}\gamma_{l}^{p}}{\gamma_{S}^{p}+\gamma_{l}^{p}}$$

(2) $$\gamma_{S-l}=\gamma_{s}+\gamma_{l}-2{\left(\gamma_{s}^{d}\gamma_{l}^{d}\right)}^{\frac{1}{2}}-2{\left(\gamma_{s}^{p}\gamma_{l}^{p}\right)}^{\frac{1}{2}}$$

(3) $$\gamma_{i}=\gamma_{i}^{d}+\gamma_{i}^{p}$$

where $\gamma_{S-l}$ is the interfacial tension between solid (*s*) and liquid (*l*); $\gamma_{i}$ is the surface energy of materials (*i*); and $\gamma_{i}^{d}$ and $\gamma_{i}^{p}$ are the dispersive and polar components of $\gamma_{i}$, respectively.

In this study, a masterbatch was prepared by concentrating GNPs in a base resin to ensure a good dispersibility of the GNPs. The interfacial tension of each material was calculated to confirm that the GNPs would come into contact with furfurylamine (FA) in the diglycidyl ether of bisphenol A-based epoxy resin (DGEBA) to enable the DA reaction of GNPs without solvent. The mechanical properties and chemical resistance of the obtained composites were enhanced through the crosslinking effect by the DA reaction. In addition, we demonstrated that the designed system can manufacture self-healing and recyclable epoxy composites through DA and retro DA reactions.

## 2. Experimental Section

### 2.1. Materials

DGEBA and 1,6-hexanediol diglycidyl ether (HDE) were supplied by Kukdo Chemical (Seoul, Korea). Epoxy equivalent weights of DGEBA and HDE were 187 and 115 g/eq respectively. Cyclohexylamine (CA), FA, and diethylenetriamine (DETA) purchased from Sigma-Aldrich (Young-in, Korea) were reagent grades (>99%). Dimethylformamide (DMF) and dimethyl acetamide (DMAc) were purchased from SK Chemical (Seongnam, Korea), and expandable graphite (EG) was purchased from Braide Graphite Group (Qingdao, China).

### 2.2. Preparation of the GNP Masterbatch

A 300-mL quartz cup containing 0.1 g of EG was placed at the center of a rotating disk in a conventional microwave oven (700 W, LG Electronics, Seoul, Korea). After 5 min of nitrogen purging, the EG was irradiated by microwave radiation for 90 s. A total of 1 g of EG and 1 L of DMF were mixed in a 2-L beaker, and the mixture was sonicated for 1 day. After sonication, the mixture was centrifugated at 3000 rpm for 45 min [37]. The supernatant obtained after centrifugation and 100 g of base resin were mixed in a round-bottom flask and magnetically stirred for 1 h. Concentrated GNPs in epoxy resin were formed after the removal of DMF at 160 °C via evaporation. The supernatants were repeatedly mixed with the concentrated GNPs in the base resin until the GNP content of the masterbatch reached 2 wt %.

### 2.3. Preparation of GNP/EP Nanocomposites

The GNP content of the GNP/EP nanocomposites varied from 0 to 1 wt % by diluting the masterbatch with neat epoxy resin. The epoxy resin containing the masterbatch and hardener were mixed at a stoichiometrically equivalent ratio at room temperature (RT). Then, the mixture was poured into a Teflon mold and cured at 70 °C for 1 day. The sample is denoted as GEN X-YZ, where “X” indicates the wt % of GNPs in the GNP/EP nanocomposite and “YZ” denotes the hardener. Sample codes and compositions of the GNP/EP nanocomposites are given in Table S1.

### 2.4. Measurements

Raman spectra were recorded with a micro-Raman spectrometer (Nanofinder 30, Tokyo, Tokyo Instruments Inc.), using a 532-nm-wavelength laser. High-resolution transmission electron microscopy was performed with a JEM 2010 system (JEOL, Akishima, Tokyo, Japan). X-ray photoelectron spectroscopy (XPS, Theta Probe AR-XPS System, Thermo Fisher Scientific, Waltham, MA, USA) was employed to analyze the composition of the GNP surface. Fourier transform infrared (FT-IR) spectra were recorded between 4000 and 400 cm^−1^ at a resolution of 4 cm^−1^ in 50 scans using an FT/IR-300E apparatus (Jasco, Easton, Easton, MD, USA). The samples were prepared by dropping the solution onto pure KBr pellets. A Phoenix150 apparatus (SEO, Suwon, Korea) was used to perform contact angle measurements. The static contact angles of liquids were measured by depositing a drop of 3–5 μL on the substrate, and the values were recorded as the tangent normal to the drop at the intersection between the liquid and the solid surface. The images were captured within 30 s of drop deposition, and the reported contact angles are the average of at least five tests performed at different locations on the surface. Differential scanning calorimetry (DSC) measurements were performed with a Q20 apparatus (TA INST., New Castle, DE, USA). DSC thermograms were recorded for all of the synthesized polymers, which were sealed in aluminum pans in a dry nitrogen atmosphere, with an empty aluminum pan as a reference. The scanning was performed at a rate of 10 °C/min. The gel fraction and swelling ratio of the samples were determined by soaking the samples in DMAc for 48 h at RT. The gel fraction and swelling ratio were calculated using the following equations:

(4) $$Swelling\;ratio=W_{s}/W_{i}$$

(5) $$Gel\;fraction=W_{d}/W_{i}\times 100$$

where $W_{i}$ is the weight of the sample before swelling, $W_{s}$ is the weight of the insoluble polymer in DMAc, and $W_{d}$ is the weight of the dried swollen sample at 110 °C. Dynamic mechanical analysis was performed using a Q800 apparatus (TA INST., New Castle, DE, USA). The scanning was performed at a rate of 5 °C/min under a nitrogen atmosphere. Dumbbell specimens with a width of 5 mm and a length of 20 mm were obtained by curing after the casting of GNP/EP nanocomposites in a dumbbell-shaped mold. A tensile test of the composites was performed using a LLOYD LR5K PLUS instrument at a crosshead speed of 1 mm/min at RT. Rheological experiments were performed using an AR2000 instrument (TA INST., New Castle, DE, USA), with decreasing frequency sweeps of 100–0.1 rad/s at a constant strain of 1% and various temperatures. A scanning electron microscope (SEM) (AIS2100C; Seron Tech Inc., Uiwang, Korea) was used for the scratch healing test, where a razor blade was applied to produce cuts in the middle of the films.

## 3. Results and Discussion

### 3.1. Characterization of GNPs in the Masterbatch

**Figure 1:**
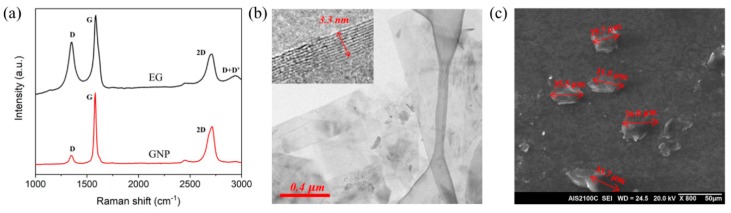
Raman spectra (**a**), HR-TEM image (**b**), and SEM image (**c**) of the graphene nanoplates (GNPs).

The quality of the fabricated GNPs is determined by the number of layers and surface defects. Raman spectroscopy and TEM imaging are useful tools for characterizing GNPs. The Raman spectra of the EG and GNPs are shown in Figure 1a. The ratio of the D to G band intensities, shown in Figure 1a, is related to the defect concentration of the GNP surface due to the overall movement of the *sp2* carbon atom in the basal plane [38,39]. The $I_{D}/I_{G}$ value for the GNPs was 0.14, which is lower than that of the EG, the starting material employed for preparing GNPs in this study, due to the reduction via microwave irradiation. The ratio of the 2D to G band intensities is also used to evaluate the quality and thickness of the GNPs. Green and Hersam reported that as the thickness of a GNP increases, $I_{2D}/I_{G}$ decreases from a maximum of 2.1 ± 0.2 for a single-layer GNP to 0.8 ± 0.1 for a quadruple-layer GNP [40]. The $I_{2D}/I_{G}$ value for the GNPs was calculated to be 0.53, corresponding to approximately 7 layers. The inset in Figure 1b displays the thickness of the GNPs, confirming a GNP layer number of approximately 10, which is close to the value calculated from $I_{2D}/I_{G}$ in the Raman spectrum. On average, the lateral size of the GNPs was 33.2 μm (Figure 1c). The GNPs in the masterbatch in this study showed excellent storage stability, without any precipitation for two months.

### 3.2. DA Reaction in the Preparation of GNP/EP Nanocomposites

Before confirming the DA reaction in the GNP/EP nanocomposites, XPS spectra were obtained to confirm the DA reaction of the GNPs with FA. GNPs and FA were allowed to react at 70 °C for 1 day, washed 3 times with acetone, and then dried at 70 °C for 1 day. The obtained GNPs were designated as modified GNPs. As shown in Figure S1, both the GNPs and modified GNPs exhibited C 1s and O 1s peaks at 284.6 and 532.5 eV, respectively [41]. A new peak at 399.5 eV for N 1s was observed in the modified GNPs, which was attributed to the DA reaction of the GNPs and FA [41]. Thus, it is postulated that the DA reaction can occur between the GNPs and FA.

**Figure 2:**
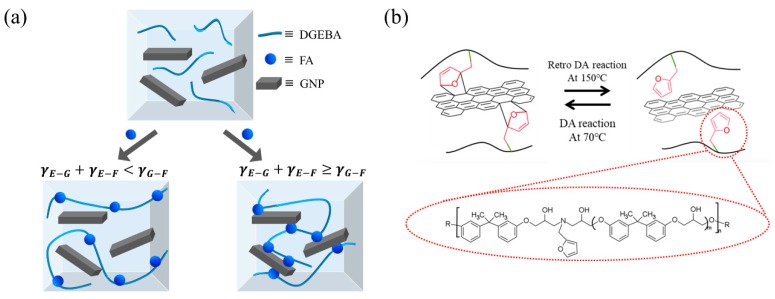
Schematics of two different dispersion behaviors in the system (**a**), and Diels-Alder (DA) and retro DA reactions between the GNPs and furfurylamine (FA) in the graphene-nanoplate/epoxy (GNP/EP) composites (**b**).

**Figure 3:**
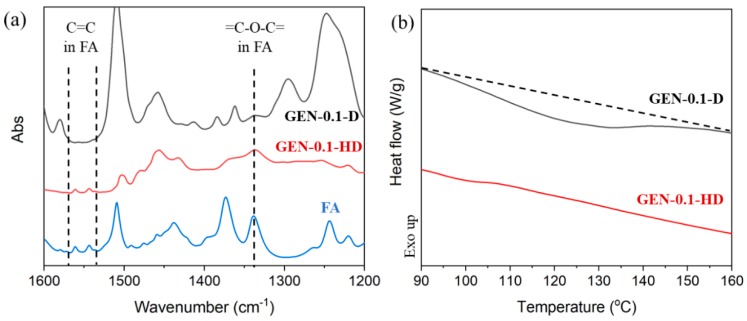
FT-IR spectra (**a**) and DSC thermograms (**b**) of GEN-0.1-D and GEN-0.1-HD.

Figure 2a illustrates the behavior of FA with different interfacial tensions in the mixture. The GNPs and FA can be close to each other when the relationship of interfacial tension is as follows:

(6) $$\gamma_{GF}<\gamma_{EF}+\gamma_{EG}$$

where $\gamma_{EG}$, $\gamma_{EF}$, and $\gamma_{GF}$ are the interfacial tensions between the epoxy resin and GNPs, the epoxy resin and FA, and the GNPs and FA, respectively. When this relationship is satisfied, a DA reaction can occur between the GNPs and FA since FA will be located close to the GNP surface. In a previous study, we confirmed an in-situ DA reaction between GNPs and furfuryl derivative for nanocomposites comprising polyurethane and GNPs [42]. The surface tension values and their components can be calculated based on the relationship between the Young and Wu equations. If the contact angles of at least two liquids with known dispersion and polar components are determined for a solid surface, then $\gamma_{s}^{d}$ and $\gamma_{s}^{p}$, as well as the surface tension of the solid, can be calculated. Using these parameters, the interfacial tension between two materials can be calculated using the Wu or Owen-Wendt equation. The obtained surface and interfacial tension values are shown in Table S2 and Table 1. The calculated interfacial tensions for the DGEBA systems, $3.54\left(\gamma_{GF}\right)<5.35\left(\gamma_{EF}\right)+7.95\left(\gamma_{EG}\right)$, satisfy Equation (6), whereas those for the HDE systems, $3.54\left(\gamma_{GF}\right)>1.58\left(\gamma_{EF}\right)+0.57\left(\gamma_{EG}\right)$, do not satisfy Equation (6). Thus, the DA reaction can occur in the DGEBA curing process, whereas it is unlikely to occur in the HDE curing process. The FT-IR and DSC results confirm this assumption, as shown in Figure 3.

The opening reaction of the oxirane ring in DGEBA upon curing was confirmed by FT-IR spectroscopy, as shown in Figure S2. After curing, the peak intensity at 915 cm^−1^ due to the oxirane ring in DGEBA was substantially reduced by the reaction [5,10,12,15,16]. In Figure 3a, FA shows characteristic peaks at 1530–1570 and 1339 cm^−1^ due to double bonds and divinyl alkyl ether bonds, respectively. After the DA reaction, the peak intensity of the furfuryl group is reduced [43]. The C=C and =C–O–C= bonds in FA disappeared in GEN-0.1-D, while the peaks in GEN-0.1-HD remained. Figure 3b shows the DSC thermograms of GEN-0.1-HD and GEN-0.1-HD. The endothermic peak at approximately 90–160 °C in GEN-0.1-D is attributable to the retro DA reaction [5,9,10,11,15,16,17]. Thus, it is postulated that the DA reaction occurred in the DGEBA systems but not in the HDE systems.

**Figure 4:**
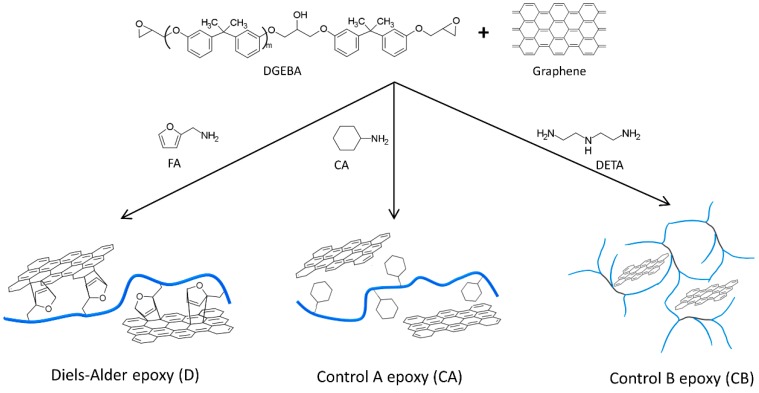
Schematic of the three types of GNP/EP nanocomposite systems.

Figure 4 illustrates the three types of GNP/EP nanocomposite systems investigated in this work. The DGEBA in the Diels-Alder epoxy resin (D system) can form a crosslinking structure through the DA reaction with FA when GNPs are added. Control A epoxy resin (CA system) was prepared using CA as the hardener instead of FA. CA was selected as a hardener because it can synthesize a linear backbone but does not contain diene. Hence, in the CA system, DGEBA cannot react with GNPs via the DA reaction, and the CA system can be compared with the D system. However, comparing the CA system and the D system with respect to the recycling of the crosslinked resins is not appropriate because of the linear backbone of the CA system. Therefore, control B epoxy resin (CB system) was manufactured using DETA, a commonly used crosslinking agent, as the hardener, and the CB system was compared with the D system to confirm the recyclability by the retro DA reaction.

**Figure 5:**
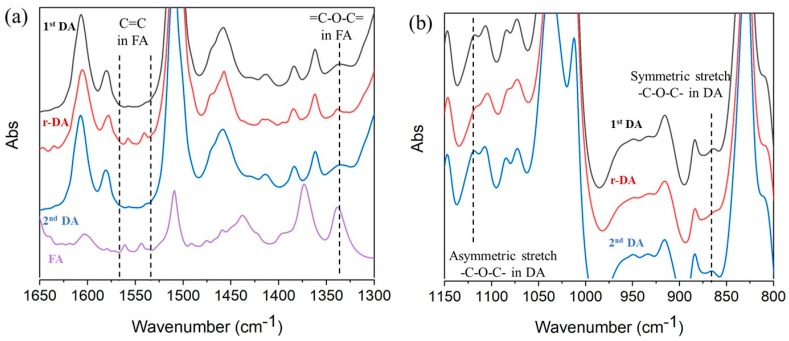
FT-IR spectra of GEN-0.1-D at various temperatures for wavenumbers ranging from 1650 to 1300 cm^−1^ (**a**) and 1150 to 800 cm^−1^ (**b**).

One double bond and a dialkyl ether exist in the DA linkage formed by the GNPs and FA. Meanwhile, when the retro DA reaction occurs, a conjugated C=C bond and a divinyl alkyl ether in FA are formed. Figure 5 shows the FT-IR spectra of GEN-0.1-DE at various temperatures. The FT-IR spectrum of GEN-0.1-DE at RT is denoted as the 1st DA. The same sample was heated to 150 °C for 30 min. The recorded spectrum is denoted as the r-DA. Then, the r-DA sample was left at 70 °C for 1 day. The final dataset was denoted as the 2nd DA. The C=C bond in the FA linkage (1530–1570 cm^−1^) was not observed in the 1st or 2nd DA. However, this peak was observed at an elevated temperature of 150 °C. The =C–O–C= peak (1339 cm^−1^) of the 1st and 2nd DA was smaller than that of the r-DA. The asymmetric and symmetric stretching of–C–O–C–in the DA linkage (1120 and 863 cm^−1^) decreased in the r-DA [44]. This change in peak intensity indicates that the DA linkage is maintained at RT and 70 °C, while the reverse reaction proceeds at 150 °C. Thus, it was confirmed that the DA reaction proceeded well during the epoxy curing process.

**Figure 6:**
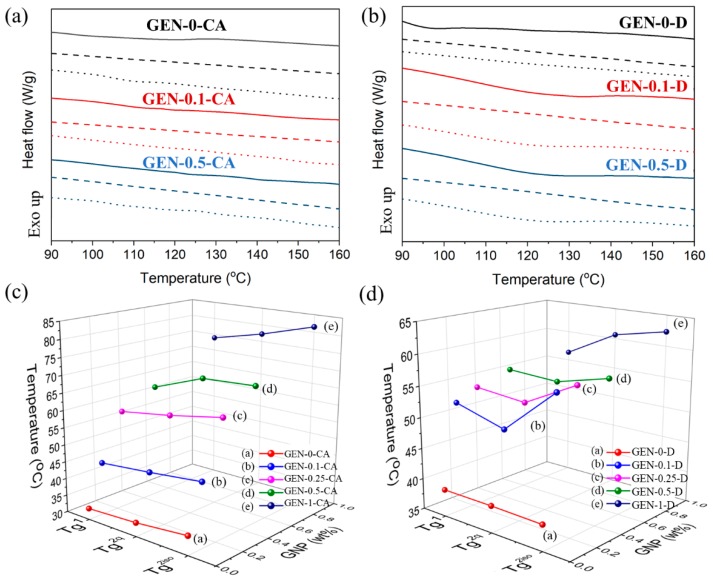
DSC thermogram of the control A epoxy resin (CA) and Diels-Alder epoxy resin (D systems). (**a**) and (**b**) present the heat flows of the composites (solid line: first scan, dashed line: second scan after the quenching process, dotted line: second scan after isothermal processing at 70 °C for 1 day). (**c**) and (**d**) display the changes in *T*_g_ based on the measurement method and GNP content (*T*_g_^1^: *T*_g_ for the first scan, *T*_g_^2q^: *T*_g_ for the second scan after the quenching process, *T*_g_^2iso^: *T*_g_ for the second scan after the isothermal process).

The endothermic peak of the DSC thermogram is often used as evidence of a retro DA reaction [5,9,10,11,15,16,17]. A secondary scan was performed using two methods to identify the endothermic peak due to the retro DA reaction. For the first secondary scan method, the sample is cooled to 0 °C immediately after the first scan, followed by a secondary scan. For the second secondary scan method, the secondary scan is performed after heat treatment in an oven at 70 °C for 1 day. The original DSC thermogram data are shown in Figure S3. Figure 6a,b presents the DSC thermograms of CA and D systems with various GNP contents. The CA system does not exhibit an endothermic peak because there was no functional group for the DA reaction. For the D systems containing GNPs, endothermic peaks due to the retro DA reaction were observed at 100~160 °C in the first and second scans after isothermal processing at 70 °C for 1 day. However, endothermic peaks due to the retro DA reaction were not observed for the quenched samples. DSC thermograms showing endotherms due to the retro DA reaction after repeated tests are shown in Figure S4. Endotherms due to the retro DA reactions were observed in the repeated tests, and thus, the reversibility of thermoset GEN-0.5-D was confirmed.

Therefore, it is postulated that the DA and retro DA reactions occur repeatedly in GNP/EP composites containing GNPs and FA. In Table 2**,** the efficiencies obtained from the enthalpy change in the endothermic peak of the D systems are given. As the GNP content increases, the efficiency of the DA reaction decreases due to the increased crosslinking density. Figure 6c,d and Table 3 show the *T*_g_ changes for the CA and D systems, respectively, based on the measurement method and GNP content. The *T*_g_ value of the CA and D systems increased with increasing GNP content. Generally, in well-dispersed GNP composites, the large interfacial surface area formed by the nanofiller affects the behavior of the surrounding polymer matrix, which reduces the mobility of the polymer chain, thereby increasing *T*_g_ [45,46]. Although the *T*_g_^2q^ values of the DE systems were lower than *T*_g_^1^ and *T*_g_^2iso^, the increase in *T*_g_ in the D system was limited by the imparted mobility of the polymer chain due to reversible crosslinking through the retro DA reaction.

### 3.3. Thermal Self-Healing Properties of GNP/EP Nanocomposites

**Figure 7:**
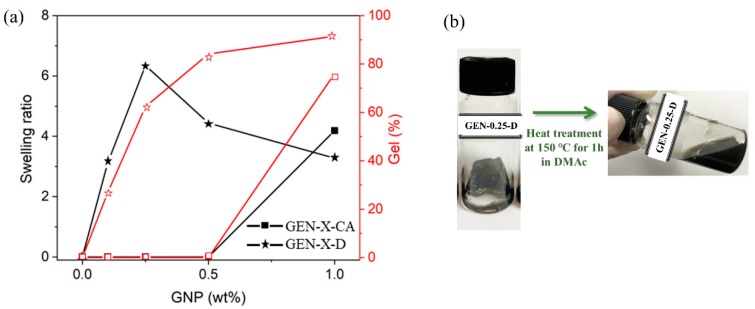
(**a**) Swelling test results for composites of the CA and D systems, (**b**) Digital image of GEN-0.25-D after heat treatment in DMAc.

The D system formed a crosslinking structure through the DA reaction, which can be confirmed via a swelling test. Figure 7a shows the results of the swelling test. Nanocomposite specimens were soaked with DMAc at RT for two days. Most GEN-X-CA composites are soluble in DMAc due to its linear structure, and a gel was observed only for GEN-1-CA. The gel observed for GEN-1-CA was attributed to the crosslinking of the CA system by reactions with functional groups on the GNP surface, as confirmed by the XPS data shown in Figure S1 for the GNPs. The functional groups of GNPs in the form of carboxylic acid, hydroxyl, and epoxide have been demonstrated by XPS in previous studies [47,48], Meanwhile, all of the D system composites produced a gel. The gel content of GEN-0.5-D is 84%, whereas the gel content of GEN-1D is 91%. The D system nanocomposites with a higher GNP concentration have a higher crosslinking density than D system nanocomposites with a low GNP concentration. The swelling ratio exhibits a maximum for GEN-0.25D due to its low crosslinking density. Furthermore, the internal space in the network structure with low-crosslinking-density composites is sufficiently large to trap the solvent. For high-GNP-concentration composites, the crosslinking density is increased; hence, the internal space in which the solvent is trapped is reduced, and the swelling ratio decreases [49]. The content or swelling ratio of the gel formed by the DA reaction can be changed by the decrease in crosslinking density caused by the retro DA reaction at high temperatures. Figure 7b shows that the solid specimen transforms into a fluid after the heat treatment of GEN-0.25-D in DMAc. The gel that swelled on GEN-0.25-D was completely soluble with DMAc after heat treatment at 150 °C for 1 h, proving that the retro DA reaction occurred at 150 °C.

**Figure 8:**
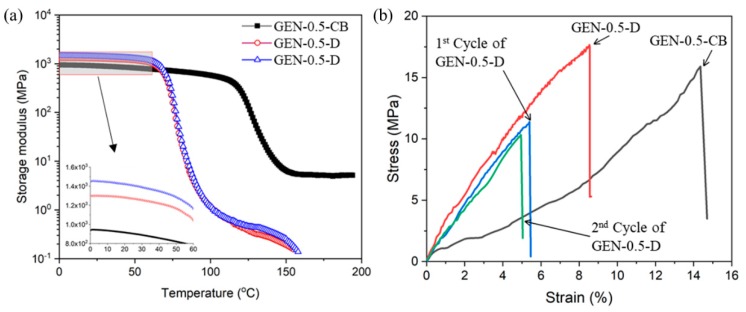
(**a**) Storage modulus of control B epoxy resin (CB) and D system composites, (**b**) Repeated recycling test results for GEN-0.5-CB and GEN-0.5-D.

The dynamic mechanical properties of the D and CB systems are illustrated in Figure 8a. The storage modulus (G’) versus temperature (T) curve of the nanocomposites in the CB system is typical for a crosslinked thermoset. Both composites show clear transitions from a glassy to a rubbery state, and the G’ value of the D system composites in the glassy state gradually increases as the GNP content increases, due to crosslinking between the GNPs and DGEBA via the DA reaction. The composites of the D system show significantly lower glass transition temperatures in comparison with those for the CB system due to the retro DA reactions at elevated temperatures. Figure 8b shows stress–strain curves for GEN-0.5-D and GEN-0.5-CB. The tensile modulus of GEN-0.5-D is larger than that of GE-0.5-CB due to crosslinking between the GNPs and the epoxy resin in the GEN-0.5-D. In the recycling tests for GEN-0.5-D, recycled specimens were successfully prepared by compression molding of powdered specimens with a hot press after the tensile test. However, powdered specimens of GEN-0.5-CB could not be remolded after the tensile test. GEN-0.5-D exhibited a tensile strength of 17.44 MPa at an elongation of 8.51%. In the recyclability test, the tensile strength of GEN-0.5-D recovered by 65% in the first cycle, and in the second cycle, a recovery of 60% was observed compared with the original GEN-0.5-D value. Water condensation of DA adducts may be one of the probable reasons for the limited rework ability after processing at T > 120 °C as reported by Cheng and Huber [50]. 

**Figure 9:**
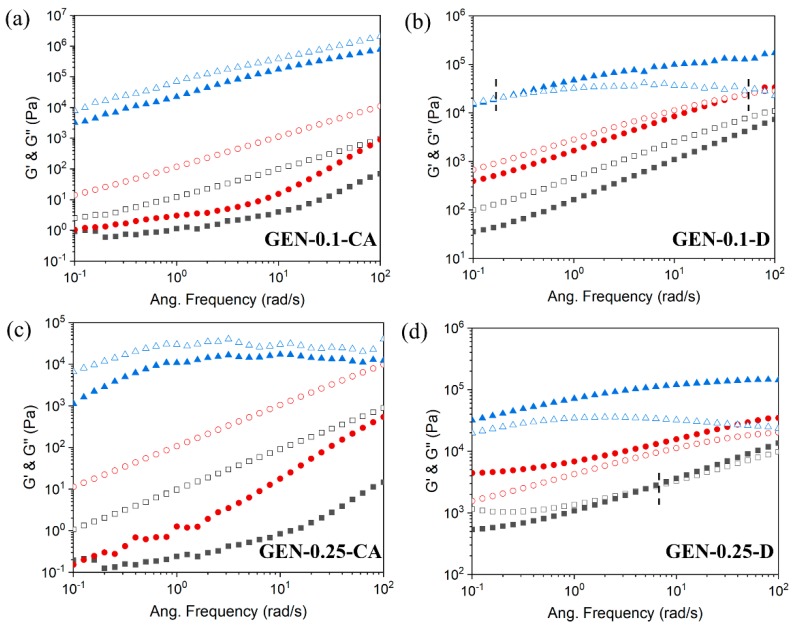
Storage (filled symbols) and loss (open symbols) moduli for composites of the CA and D systems at 80 °C (▲), 120 °C (●), and 150 °C (■).

Rheological measurements of thermosetting resins provide insights into chemical and physical changes caused by reactions occurring at specific temperatures. Figure 9 shows the changes in G’ and G” at various temperatures over different frequency sweeps. The composites of CA systems have a loss modulus that is higher than the storage modulus regardless of the GNP content or measurement temperature. These composites show a liquid-like behavior. Meanwhile, for the D system composites, the loss modulus intersects with the storage modulus, and the composite can flow beyond the gel point. The crossover of G’ and G’’ occurs at 0.20 and 50.12 rad/s for 80 and 120 °C in GEN-0.1-D, with fluidity occurring at 150 °C. The relaxation times obtained from the frequency values for the crossover of G’ and G” are given in Table S3. In general, the curing process of the thermoset resin caused the polymer to exhibit glassy or liquid-like behavior based on the gel point. Considering that the crosslinking of the DA reaction is due to a dynamic covalent bond, the breakdown and reforming of the structure over different timescales can be identified by the temperature-dependent behavior [51].

**Figure 10:**
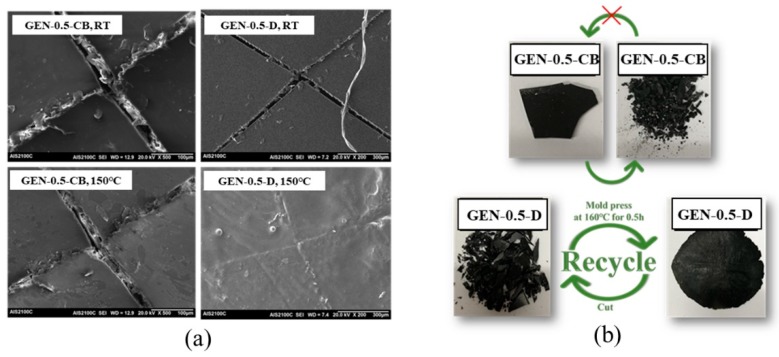
(**a**) SEM images of the scratch healing test for GEN-0.5-CB and GEN-0.5-D, and (**b**) reprocessing of the composites in small pieces with an external force of less than 3 MPa and heat treatment at 160 °C for 0.5 h.

A scratch on the surface of the polymer specimens can be healed when reversible or exchange reactions are introduced in the polymer. The specimens were scratched with a razor blade and then gold-coated. The thermal self-healing properties of the nanocomposites were investigated after heat treatment at 150 ℃ on a heating plate for 1 h. Figure 10a shows representative SEM images of the scratched specimens before and after the self-healing tests. The scratch remained in GEN-0.5-CB, while that of GEN-0.5-D gradually disappeared within 1 h. The scratch healing ability was imparted to GEN-0.5-D by the retro DA reactions. We infer that the small pieces can be reprocessed at elevated temperatures via the retro DA reaction. To confirm the reprocessing ability, GEN-0.5-CB and GEN-0.5-D were pulverized, and then the samples were placed between two steel plates and heat treated at 160 °C for 0.5 h with an external force of less than 3 MPa. Figure 10b shows the reprocessed samples before and after reprocessing. The pieces of GEN-0.5-CB remain after the heat treatment; however, a film was obtained from the pulverized GEN-0.5-D sample after heat treatment under the same conditions employed for GEN-0.5-CB.

## 4. Conclusions

In this study, thermally remendable GNP/EP nanocomposites were successfully synthesized via an in-situ DA reaction between GNPs and FA during the epoxy curing process. The interfacial tension between each material was calculated, and the conditions for the DA reaction between the GNPs and FA during the DGEBA curing process were determined. The DA and retro DA reactions were confirmed through FT-IR spectroscopy, DSC thermography, and rheological measurements. Due to the reversible crosslinked structure in this system, scratch healing and reprocessing ability were achieved at 150 °C. The crosslinked structure of D system composites was exhibited at RT; however, thermal molding for recycling was possible at high temperatures due to the reversibility of the DA reaction.

## Figures and Tables

**Table 1:** Interfacial tension between the constituents of the GNP/EP nanocomposites.

Materials	Interfacial Tension (dyne/cm)	
	Wu	Owen-Wendt
DGEBA/GNP	7.95	4.06
DGEBA/FA	5.35	2.79
HDE/GNP	0.57	0.29
HDE/FA	1.58	0.79
GNP/FA	3.54	1.79

**Table 2:** Enthalpy changes in the GEN-X-D composites.

Sample	1st ΔH (J/g)	2nd ΔH (J/g)	Efficiency ^a^ (%)
GEN-0-D	-	-	-
GEN-0.1-D	3.5	2.4	68.6
GEN-0.25-D	5.7	3.2	56.4
GEN-0.5-D	6.8	2.9	41.9
GEN-1-D	4.8	1.5	31.5

^a^ Efficiency (%) = $\left(2^{nd}\Delta H/1^{st}\Delta H\right)\times 100$.

**Table 3:** Summary of Tg based on the measurement method and GNP content.

Sample	*T*_g_^1^ (°C)	*T*_g_^2q^ (°C)	*T*_g_^2is^° (°C)	Sample	*T*_g_^1^ (°C)	*T*_g_^2q^ (°C)	*T*_g_^2iso^ (°C)
GEN-0-CA	33.3	34.0	35.59	GEN-0-D	39.4	39.3	39.1
GEN-0.1-CA	45.3	46.7	58.4	GEN-0.1-D	52.7	50.4	57.6
GEN-0.25-CA	58.5	60.7	63.6	GEN-0.25-D	54.3	53.5	57.8
GEN-0.5-CA	63.1	68.5	69.3	GEN-0.5-D	55.8	55.4	57.4
GEN-1-CA	73.9	77.2	81.5	GEN-1-D	56.4	60.6	62.3

## Supplementary Materials

The following are available online at https://www.mdpi.com/2073-4360/11/6/1057/s1. Table S1: Sample code and compositions of the GNP/EP nanocomposites; Table S2: Components of the surface tensions (20 °C); Table S3: Relaxation times of D system nanocomposites; Figure S1: XPS spectra of GNPs and modified GNPs; Figure S2: FT-IR spectra of GEN-0.1-D and DGEBA; Figure S3: DSC thermograms of the CA and D systems of different GNP contents: (a) First scan; (b) Second scan after quenching at 0 °C; (c) Second scan after the isothermal condition at 70 °C for 1 h; Figure S4: DSC thermograms of the D system composites: (a) third scan; (b) repeated endothermic peak of GEN-0.5-D.
