# Supplementary material for: Thermally Healable and Recyclable Graphene-Nanoplate/Epoxy Composites Via an In-Situ Diels-Alder Reaction on the Graphene-Nanoplate Surface
Source: Polymers (Basel). 2019 Jun 18;11(6):1057. doi: 10.3390/polym11061057 (PMC6630711; doi:10.3390/polym11061057)
Supplement: Supplementary file 1 [file polymers-11-01057-s001.pdf]

Supplementary Materials

Thermally healable and recyclable graphene-nanoplate/epoxy composites via an *in-situ* Diels–Alder reaction on the graphene-nanoplate surface

Cho-Rong Oh[1,†], Dae-Il Lee[1,†], Jun-Hong Park[2,*], and Dai-Soo Lee[1,*]

[1] Division of Semiconductor and Chemical Engineering, Chonbuk National University, Baekjedaero 567, Jeonju 54896, Republic of Korea; ohcho38@naver.com (C.R.O.); ldi4084@naver.com (D.I.L.)

[2] R & D Center, Lotte Advanced Materials, Sandan-ro 334-27, Yeosu, 59616, Republic of Korea

* Correspondence: daisoolee@jbnu.ac.kr (D.S.L.), jh711.park@lottechem.com (J.H.P.); Tel.: +82-63-270-2310 (D.S.L.); +82-61-689-1731 (J.H.P.)

† These authors contributed equally to this work.

Table S1. Sample code and compositions of the GNP/EP nanocomposites

Sample code	Composition (wt%)					
	DGEBA	HDE	CA	FA	DETA	GNP
GEN-0-CA	80.54	0	19.46	0	0	0
GEN-0.1-CA	80.46	0	19.44	0	0	0.10
GEN-0.25-CA	80.34	0	19.41	0	0	0.25
GEN-0.5-CA	80.14	0	19.36	0	0	0.50
GEN-1-CA	79.74	0	19.26	0	0	1
GEN-0-D	80.87	0	0	19.13	0	0
GEN-0.1-D	80.79	0	0	19.11	0	0.1
GEN-0.25-D	80.67	0	0	19.08	0	0.25
GEN-0.5-D	80.46	0	0	19.04	0	0.50
GEN-1-D	80.06	0	0	18.94	0	1
GEN-0.1-HD	0	77.29	0	22.61	0	0.10
GEN-0.5-CB	90.41	0	0	0	9.09	0.50

Table S2. Components of the surface tensions (20 °C)

Materials	γ_i (dyne / cm)	γ_i^d (dyne / cm)	γ_i^p (dyne / cm)
Toluene[a]	28.5	27.2	1.3
Deionized water[a]	72.8	21.8	51
Slide glass	72.8	29.2	43.6
Teflon film	36.1	27.3	8.8
GNP	39.9	29.9	9.9
DGEBA	68.7	54.7	14.0
HDE	39.6	32.5	5.8
FA	48.0	42.6	5.4

a) γ_i, γ_i^d, and γ_i^p of toluene and deionized water were obtained from literatures [1,2].

The components of the surface tension for the slide glass and the Teflon film were determined using the equations (1) and Young's equation with the contact angles of two different liquids, deionized water and toluene, to obtain the surface tension of FA. The components of surface tensions for GNP and DGEBA were also obtained by the same processes. The components of surface tension obtained are summarized above in Table S2.

Table S3. Relaxation times of D system nanocomposites

Sample code	Cross-over of G′ and G′′ (rad/s)	Cross-over of G′ and G′′ (Hz)	Relaxation time (s)
GEN-0.1-D (80 ℃)	0.20	0.03	31.42
GEN-0.1-D (120 ℃)	50.12	7.98	0.13
GEN-0.25-D (150℃)	5.01	0.80	1.25

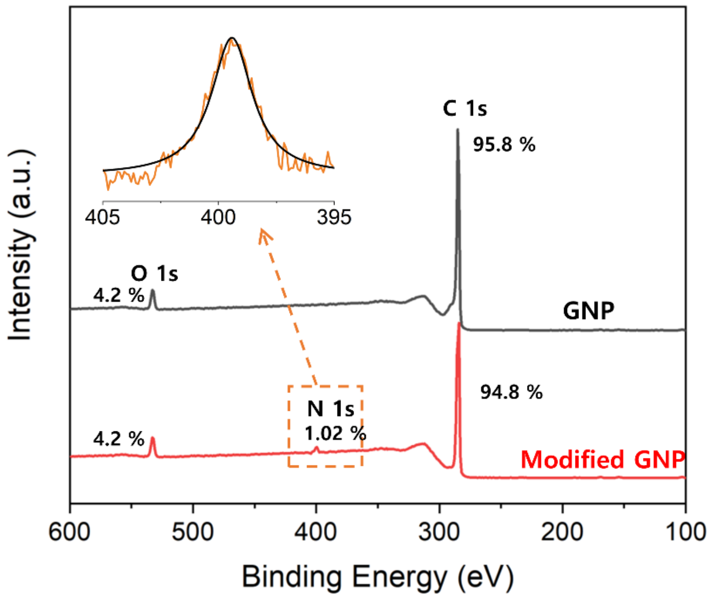

Figure S1. XPS spectra of GNP and modified GNP.

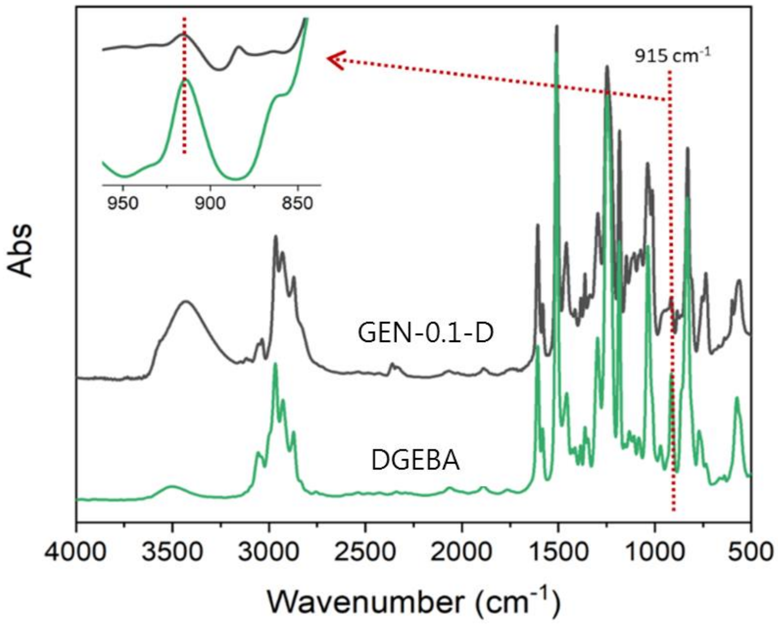

Figure S2. FT-IR spectra of GEN-0.1-D and DGEBA.

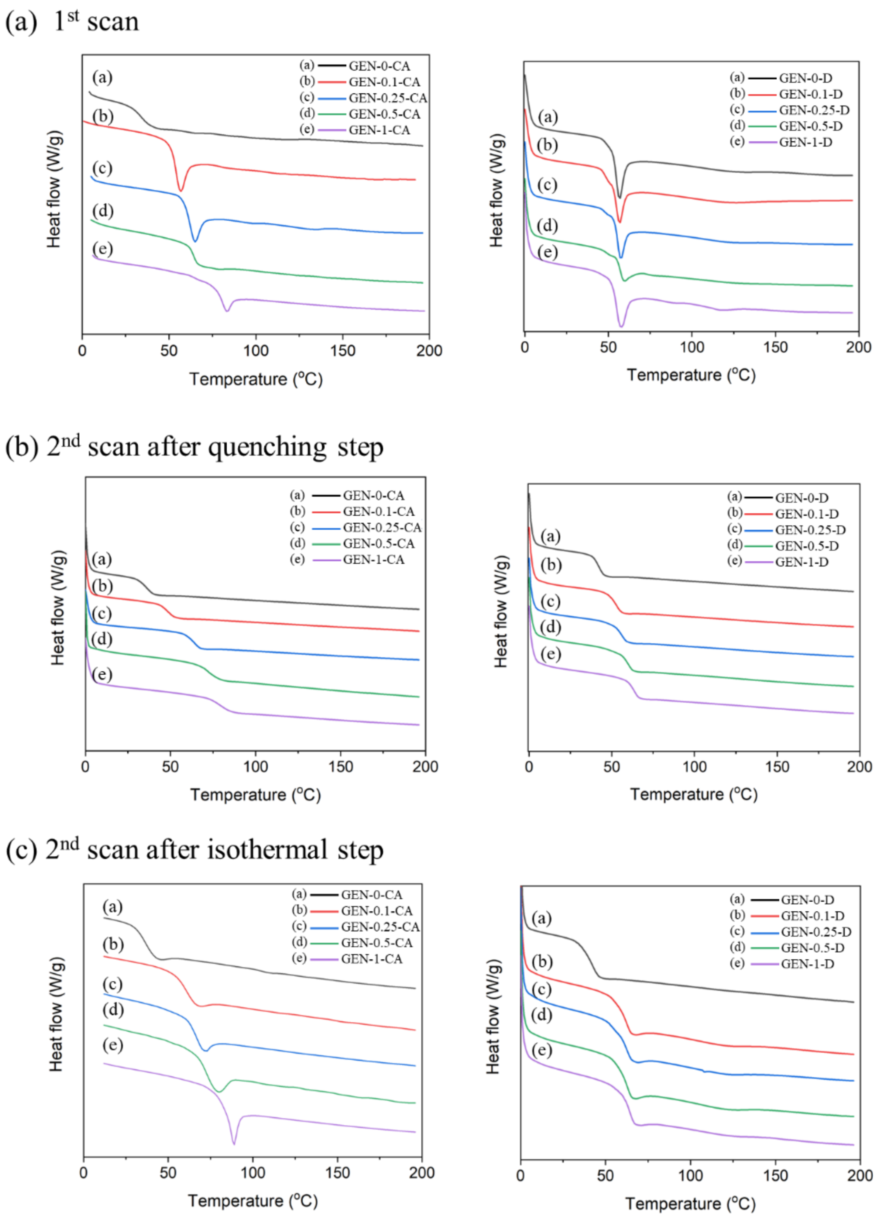

Figure S3. DSC thermograms of the CA and D systems of different GNP contents: (a) First scan; (b) Second scan after quenching at 0 °C; (c) Second scan after the isothermal condition at 70 °C for 1h.

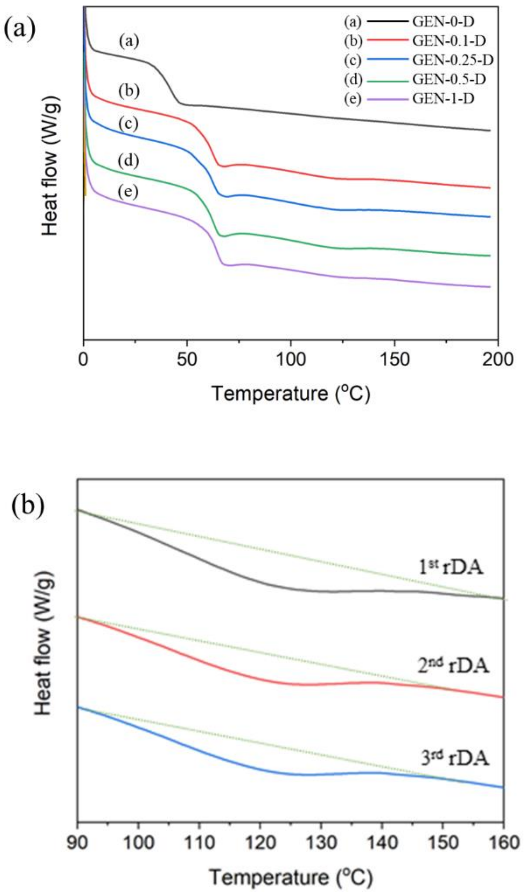

Figure S4. DSC thermograms of the D system composites: (a) third scan; (b) repeated endothermic peak of GEN-0.5-D.

Reference

1. Moncayo, D.; Buitrago, G.; Algecira, N. The surface properties of biopolymer-coated fruit: A review. *Ingeniería e Investigación* **2013**, *33*, 11-16.
2. Van Oss, C.J.; Ju, L.; Chaudhury, M.K., Good, R.J. Estimation of the polar parameters of the surface tension of liquids by contact angle measurements on gels. *J. Colloid Interface Sci.* **1989**, *128*, 313-319.
